# The Coordination Between B Cell Receptor Signaling and the Actin Cytoskeleton During B Cell Activation

**DOI:** 10.3389/fimmu.2018.03096

**Published:** 2019-01-09

**Authors:** Jingwen Li, Wei Yin, Yukai Jing, Danqing Kang, Lu Yang, Jiali Cheng, Ze Yu, Zican Peng, Xingbo Li, Yue Wen, Xizi Sun, Boxu Ren, Chaohong Liu

**Affiliations:** ^1^Department of Microbiology, School of Basic Medicine, Tongji Medical College, Huazhong University of Science and Technology, Wuhan, China; ^2^Wuhan Children's Hospital, Tongji Medical College, Huazhong University of Science and Technology, Wuhan, China; ^3^Department of Immunology, School of Medicine, Yangtze University, Jingzhou, China; ^4^Clinical Molecular Immunology Center, School of Medicine, Yangtze University, Jingzhou, China

**Keywords:** actin, B cell, BCR signaling, membrane-associated antigen, B cell activation

## Abstract

B-cell activation plays a crucial part in the immune system and is initiated via interaction between the B cell receptor (BCR) and specific antigens. In recent years with the help of modern imaging techniques, it was found that the cortical actin cytoskeleton changes dramatically during B-cell activation. In this review, we discuss how actin-cytoskeleton reorganization regulates BCR signaling in different stages of B-cell activation, specifically when stimulated by antigens, and also how this reorganization is mediated by BCR signaling molecules. Abnormal BCR signaling is associated with the progression of lymphoma and immunological diseases including autoimmune disorders, and recent studies have proved that impaired actin cytoskeleton can devastate the normal activation of B cells. Therefore, to figure out the coordination between the actin cytoskeleton and BCR signaling may reveal an underlying mechanism of B-cell activation, which has potential for new treatments for B-cell associated diseases.

## Introduction

B cells are an important set of immunocompetent cells. They have two main functions: 1. to participate in the immune response directly by humoral immunity (antibody production) (1), and 2. to participate in the T-cell immune response as specific antigen presenting cells that selectively capture and present antigens to T cells (2, 3). These two functions of B cells are achieved through activation of the surface BCR. Just like the TCR/CD3 complex, the BCR is also a complex of oligomers (4). It has been verified that the BCR is composed of the surface membrane immunoglobulin (mIg) including IgM and IgD in the naive B cell and IgG in the memory B cell, which functions as the antigen-binding part, and the signaling components consisting of non-covalently associated Igα and Igβ (CD79a and CD79b) heterodimer (4, 5). Both mIg and Igα/β contain transmembrane heavy chains with the cytoplasmic tails extending into the cell cortex (6). The length of the cytoplasmic tail of the antigen-binding part differs according to its isotypes. The cytoplasmic domain of mIgM and mIgD contain three amino acids, while in mIgG, the length is nearly 28 amino acids (4). The cytoplasmic tail of the signaling part contains immunoreceptor tyrosine-based activation motifs (ITAMs) (5, 7, 8), but there is no intrinsic kinase activity in BCR, and thus recruitment of the tyrosine kinase is necessary for BCR signaling. Both multivalent soluble antigens (sAg) and membrane-bound antigens (mAg) can be recognized by BCRs (9), while the mAg has a lower threshold for B-cell activation. This is consistent with the mode of *in vivo* antigen recognition which is mainly through antigen-presentation by dendritic cells, follicular dendritic cells, and macrophages (10, 11). It has been observed that monovalent mAg but not monovalent sAg can induce B-cell activation (9, 12, 13). Different from the T cell, the MHC molecular on the antigen presenting cell is not required by B cell during antigen recognition (7), so new models should be built to understand how the mAg is given the priority compared with the sAg. After effective stimulation of antigens, the tyrosines of ITAM in the BCR are phosphorylated by tyrosine kinase Lyn, one of the Src family proteins, and the spleen tyrosine kinase (Syk) (14–18). The interaction between BCR-associated Src-family kinase and CD19 results in CD19 and PI3K phosphorylation (7, 17). Signaling molecules including PLC γ and Vav are also phosphorylated and recruited through Syk (16, 19, 20). Under the catalysis of PLCγ, phosphatidylinositols releases IP3 which is important for Ca2^+^ release, and DAG which promotes the activation of PKC (21). GTPases including Ras and Rap1 are activated, and participate in the activation of MAP kinases such as JNK, Erk, and p38 (22). Activation of the BCR finally leads to B-cell proliferation and antibody production.

Disorders of BCR signaling can lead to immunological diseases. Studies have proved several diseases related with the dysregulation of the actin cytoskeleton, including the Wiskott-Aldrich syndrome (WAS), an immunodeficiency disease resulted from the deficiency of WAS protein (WASP), an important actin regulator in haematopoietic cells, or WASP interacting protein (WIP) (23–26). Diffuse large B cell lymphoma (DLBCL) has been showed highly associated with unusually high levels of phosphorylated actin binding proteins Ezrin-Radixin-Moesin (ERM) (27). The studies indicate the potential role of actin in both up-regulation and down-regulation of BCR signaling. Recent studies using biochemical or microscopy technologies have showed during B-cell activation, awell-regulated actin-cytoskeleton reorganization is required to achieve processes including receptor clustering, signaling-molecule recruitment, and B-cell morphological changes, which is in turn accurately controlled by BCR signaling. In this review, firstly we provide a glance of the structure of the actin cytoskeleton in B-cell cortex. BCR dynamics on a nanoscale is also introduced on a nanoscale. Then we discuss the potential role of actin in the initiation of BCR triggering. Later we introduce how the actin cytoskeleton participates in the formation of BCR microclusters and the immune synapse. Finally we talk about the regulation of BCR signaling on actin-cytoskeleton reorganization.

## Structure of the Cortical Actin Cytoskeleton

The cortical actin cytoskeleton also known as the cell cortex is a thin network just beneath the plasma membrane, and exists in most animal cells. It is the dominating actin structure in B cells, so the actin cytoskeleton we talk about in this review refers to the cortical actin cytoskeleton. The cortical actin cytoskeleton contains over a hundred actin-binding proteins (ABPs) (28). It is connected to the plasma membrane through several membrane-cytoskeleton linkers including myosin 1 and ERM proteins which contain three conserved and related proteins (ezrin, radixin and moesin) (28, 29), and is pulled on by myosin-2 which provides contractile stresses and thus produces the cortical tension (30, 31). Dynamic changes of actin filaments are required to achieve cell morphological changes. These processes are mediated by actin binding proteins including F-actin nucleators, regulators of actin assembly and disassembly, and actin crosslinkers (28, 32). F-actin nucleators include formins which nucleates and lengthens the linear F-actin (33), and the actin-related protein 2/3 (ARP2/3) complex which promotes the formation of branched F-actin (28, 34). The nucleators are important in regulating cortical elasticity and cortex tension through controlling the length of actin filaments, which allows cells to adapt to environments with different mechanical properties (30, 35). Regulators of actin assembly and disassembly include the capping proteins that can inhibit the growth of F-actin through binding to its barbed end. The actin-assembly promoting protein profilin, and the actin severing protein cofilin (28, 32, 36). The combined actions of these actin binding proteins produce different membrane protrusion structures (31, 37, 38), for example, lamellipodia, a sheet-like protrusive structure, is composed of branched F-actin, and filopodia which looks like a finger, is composed of linear F-actin (39, 40). Through controlling dynamic morphological changes, the actin cytoskeleton is crucial in the polarization, adhesion as well as migration of the B cell (41–44).

## BCR Dynamics during B-cell Activation

The technique of classicial biochemistry helps us to gain the information of interactions between individual signaling molecules and provides with the basis to investigate B-cell activation. However, to clarify the mechanisms underlying B-cell activation, more information of molecular dynamic changes under the correct cellular context is needed. Fortunately, new technologies combined of high or super-resolution imaging methods and fluorescence probes have provided us with a more detailed and quantitative description of the spatiotemporal dynamics of BCRs on B cell (45–47).

There have been methods to follow the lateral diffusion of membrane molecules, such as single particle tracking (SPT), which has been used as the total internal reflection microscopy (TIRFM) developed in recent years (48–50). Studies have showed that BCRs do not diffuse freely on the surface of the B cell, but were restricted within discrete confinement zones with a diameter of 40~200 nm (51, 52). The average diffusion coefficient of IgM-based BCRs is ~0.03 μm^2^/s (9, 47, 53). Besides, using direct stochastic optical reconstruction microscopy (dSTORM), it was demonstrated BCRs in the resting state existing in nanometer sized clusters called the “protein island” or the “nanocluster,” which differ in size as well as numbers of single BCR molecules (47, 54). IgM and IgD BCRs actually localize in different compartments which are class-specific, though the molecular mechanism underlying the distribution is little known (47, 55, 56). Under the restriction of BCR diffusion, only antigen-independent tonic signaling which is much lower than antigen-induced one is allowed (51, 57).

Upon the engagement of the membrane-associated antigen, the radius of the BCR nanoclusters increases, which seems to be in accordance with the growing number of the BCR in the cluster, while the density of BCR nanoclusters decreases (49, 54). Besides, BCR nanoclusters become more dispersed and the lateral mobility increases with an average speed of 0.05 μm^2^/s when stimulated by mAg (9, 53), leading to collisions between nanoclusters, which results in the formation of BCR microclusters which are composed of 50 ~ 500 single BCRs including both IgM and IgD BCRs (54, 58). Minutes after mAg stimulation, the B cell begins to spread on the antigen-associated membrane, which lasts for 5–10 min (59), at the same time, more BCR microclusters form and these microclusters move toward the center of the contact area (18), with an average speed of ~0.01 μm/s (59, 60). Then along with the followed B-cell contraction (59–61), the microclusters together form a central suprmolecular activation cluster (cSMAC) which is characterized as the core of immune synapse (IS) (61–63). As microclusters coalesce with each other, the lateral mobility of single BCR molecules in clusters decrease to 0.02 μm^2^/s, an average speed (53), similar with BCRs within nanoclusters before stimulation. The mature immune synapse takes about 10 min to assemble and is followed by BCR-antigen-complex internalization and antigen processing (64).

Multivalent sAg can induce similar dynamics of BCRs as induced by mAg. Both of the two-type antigens induce the formation of central clusters. However, the central cluster of BCRs forms at one pole when stimulated by sAg, while it forms at the center of the area contacting with antigen-associated membrane when stimulated by mAg. Besides, the morphological changes of B cell contracting after its spreading particularly take place in mAg-stimulated B-cell activation, and sAg can only induce protrusion structures at the area of BCR central cluster (41). The two-phase response is recognized as the basic morphological event during B cell activation stimulated by mAg (59, 60).

## The Actin Cytoskeleton Potentially Participates in B Cell Antigen Receptor Triggering

There are more than 100,000 BCR complexes expressed on the surface of a mature B cell (47). How these BCRs keep inactive and how they are triggered by antigens is a key question in B cell researches, but so long hasn't been well-understood. In recent years, several new models have been raised about this question. All of them focus on BCR conformation and BCR-BCR interactions which seem different between BCRs within nanoclusters and those within antigen-induced microclusters. The conformation induced oligomerization model (CIOM) suggests that in resting B cells, the majority of BCRs exists as monomers, and the activation is inhibited due to interactions between themselves which leads to blocking of the ITAM domain. Antigen-binding induced conformational changes expose the ITAM domain to recruit signaling molecules and allow BCR to form signaling-active oligomers (9, 49). This model may provide explanation for the signaling attenuation during the formation of BCR central cluster (later discussed), since both resting BCRs and BCRs within central clusters have similar lateral mobility which is controlled by the actin cytoskeleton (later discussed), suggesting the potential role of the actin cytoskeleton in the switch of BCR state between inhibition and activation. Other explanations include the dissociation activation model (DAM), which supports BCRs mainly existing as self-suppressed oligomers in resting B cells. The binding of antigen promotes dissociation of BCR olignomers and leads to BCR activation (13, 56, 65). During this process, BCR dissociation from oligomers and aggregation into larger clusters are considered as events happened at different level of size as well as time point. It was found treating B cells with only Lat-PLA induced the dissociation between BCR oligomers (Reth M. et al. unpublished data) (66), suggesting that it's likely that the disruption of the actin cytoskeleton results in BCR dissociation at the initiation of BCR activation.

## The Actin Cytoskeleton Regulates the Formation of BCR Microclusters and the Immune Synapse

BCR microclusters can also be defined as the “microsignalosomes” as they recruit intracellular signaling molecules and adaptors such as Lyn, Syk, Vav, PLC-γ 2, and CD19, and thus mediate signal transduction (18, 19, 67, 68). Later BCR microclusters together with the associated antigens aggregate into a central cluster. The central cluster acts as the core of the immune synapse and the region where the later antigen extraction takes place (59). The level of BCR signaling and the quantity of antigens which are later presented to T cells depend on the process of BCR-microcluster and immune-synapse formation (61), and the extent of B-cell activation is thus determined. To achieve maximized activation in response to different antigens, the formation of BCR microclusters and the immune synapse differs according to different antigen properties including density, valency, affinity, mobility, and the stiffness and topography of the antigen-binding substrates (49, 69–71). The mechanism underlying this adaptive capacity of B cells is the regulation of actin cytoskeleton.

### Regulation on BCR Mobility

As we have mentioned, in resting B cells, the mobility of BCRs is restricted. It has been showed that the cortical actin cytoskeleton acts as a barrier to confine BCR diffusion. The efficiency of the restriction on BCR diffusion depends on the cytoplasmic domain of Igβ on a large scale (51). Observations showed the mobility of BCRs is negatively related to the density of F-actin in the plasma membrane (51). Treating B cells with Latrunculin A leads to the disruption of the actin network, which can increase BCR diffusion (46, 51, 72). Furthermore, studies using high-speed dual-view acquisition TIRFM to observe the BCR as well as actin and ezrin simultaneously showed that ezrin together with actin formed the network which confines BCRs in nanoscale compartments. In resting B cells, ezrin in an open active conformation is associated with the actin cytoskeleton as well as integral membrane proteins through Csk-binding protein (Cbp), thus confine the diffusion of membrane proteins (73). The expression of ezrin with abnormal construct (Ez-DN) which is not able to bind to actin cytoskeleton can increase BCR mobility (53).

When BCRs bind to antigens, the actin cytoskeleton firstly undergoes a transient depolymerization (53, 58). At the same time, there is an increase in BCR diffusion (51). The disassembly of actin cytoskeleton is induced by cofilin-mediated severing and ezrin-dephosphorylation-mediated dissociation between F-actin and plasma membrane (53, 58). The disassembly of F-actin frees BCRs, both antigen-bound, and unbound ones from the barriers, which permits BCR nanoclusters to collide and interact with each other to form microclusters (53), thus amplify signaling (74). Recent studies found that lipopolysaccharide and CpG DNA, both of which are Toll-like receptor ligands, enhance BCR signaling through increasing cofilin activation (75). Increased BCR mobility primes B cells for more rapid microcluster formation when encountering antigens, which lower the threshold for B-cell activation. Soon after actin disassembly, there is a rapid reassembly of F-actin, while the structure becomes more dynamic and polarized than the pre-actived one. It's indicated that the flow of actin driven by myosin also promotes BCR microcluster formation (76). F-actin can be pushed into aster-like structures by myosin, which influences the diffusion of its binding proteins and potentially promotes them into clusters (77–79). Besides, during B cell activation, linear BCR movements at the periphery of filopodia which is defined as an actin-rich area have been observed (9), suggesting the diffusion of BCR clusters may be influenced by different F-actin-based structure.

Increased BCR mobility induced the interaction between the BCR and its co-receptor CD19. In resting B cells, the protein islands of BCR and CD19 are located in separated compartments, and the interaction between them is inhibited (46, 47, 80). In contrast to BCRs, the mobility of CD19 is not affected by the disruption of the actin cytoskeleton apparently. Instead, the lack of CD81 increases CD19 mobility when the actin cytoskeleton is disrupted (46, 47), indicating that the immobilization of CD19 was due to its existence in protein islands organized by CD81 on a large scale. A recent study has found WIP influenced CD19 diffusion through regulating CD81 expression rather than actin reorganization (81). Though the mobility of CD19 is limited, disruption of the actin cytoskeleton can initiate CD19 signaling pathway (47). These observations suggest that the actin cytoskeleton inhibits the interaction between BCRs and CD19 in the resting state mainly through the restriction of BCR mobility (64, 78, 82). The break of the barrier and the increase in BCR mobility allow the access of BCR to CD19, and thus induce CD19 signaling (47).

### Regulation on B Cell Morphology

As we have mentioned, the B cell undergoes a two-phase morphological change in response to mAg. The two-phase change depends on actin cytoskeleton remodeling. Upon antigen stimulation, breakdown of the cortical cytoskeleton is concomitant with assembly of branched F-actin at the cell periphery (58, 59). Filopodia firstly appears to contact the antigen-associated membrane. Soon after the stimulation, F-actin accumulates at the contact area particularly in the peripheral region to generate filopodia and lamellipodia which make dynamic changes between extension and contraction (41). These dendritic actin structures promote B-cell spreading which extends the contact zone between the B cell and the antigen-associated membrane. When the filopodia and lamellipodia extend, new BCR microclusters form, often at the tip of these structures, and newly formed BCR microclusters are pushed inward when the filopodia and lamellipodia contract (41, 60). Simultaneously with the formation of the dendritic actin structure, the MTOC and microtubule networks undergo reorganizations toward the contact area, which promotes BCR microclusters aggregating into the center cluster (83). This process needs the participation of dynein motor protein and IQGAP1 which can link the microtubule and the actin cytoskeleton (84), and depends on cofilin-mediated actin severing and actin-mediated B cell spreading (85). The contact area between the B cell and the antigen-associated membrane keeps increasing as actin accumulates, during a several-minute timescale which is concerned with the nature of antigens (59). F-actin accumulation is followed by a decrease in the region nearby merging BCR clusters, while the level of F-actin maintained at the periphery of the contact area (41, 58, 74). At the same time, there is a reduction in B cell membrane dynamics, accompanied with contraction rather than extension of the filopodia and lamellipodia. These finally result in the contraction of the contact zone, during which the retrograde flow of actin and the mechanical force provided by contraction leads to the aggregation of BCR microclusters and finally the formation of the BCR center cluster (59, 60).

The regulation of actin cytoskeleton on B cell morphological changes produces both positive and negative effects on BCR signaling. During B cell spreading, the B cell contacts with the antigen-associated membrane to recognize and combine as many antigens as possible, and promotes the formation of new BCR microclusters, which amplifies BCR signaling. During B-cell contraction, BCR microclusters as well as the bound antigens merge into the central cluster (59). This coalescence is associated with BCR signaling attenuation at B cell surface, which can be inhibited by blocking B cell contraction (60), suggesting BCR central cluster formation promoted by the actin cytoskeleton is a mechanism for the down-regulation of BCR signaling.

### Regulation on the Interaction Between Signaling Molecules

The actin cytoskeleton regulates the interaction between signaling molecules through its influence on the diffusion of membrane molecules. The transient disassembly and later assembly of the actin cytoskeleton apply distinct influence in different stage of BCR signaling. Dissociation of the actin cytoskeleton increases the mobility of proteins and thus promotes collisions between them (80). As mentioned above, the reorganization of the actin cytoskeleton induces the interaction between CD19 and BCRs by increasing BCR mobility. Besides, the function of the negative co-receptor CD22 on BCR is also regulated by the actin cytoskeleton. Different from CD19, the regulation is through CD22 itself, which seems be associated with a sialic acid-binding domain which was found closely correlated with CD22 lateral mobility and nanocluster organization (46). CD22 performs its inhibitory function through the recruitment of SH2-domain-containing phosphatase-1 (SHP-1) and the inositol phosphatase SHIP, downstream signaling molecule of FcγRIIB which is a negative co-receptor of BCRs, to its immune-receptor tyrosine-based inhibitory motifs (ITIMs) after BCR activation (82, 86, 87). Studies have proved that during BCR activation led by the disruption of the cortical actin, the lateral mobility of CD22 is increased and resulted in a relatively low level of BCR signaling compared with BCR crosslinking (41, 46), suggesting that the negative function of CD22 on BCR signaling is partly inhibited by the actin cytoskeleton. Besides the co-receptor of BCRs, the actin cytoskeleton regulates dynamics of lipid rafts through the actin-binding protein ezrin (72, 73), and thus influence the interaction between BCRs and various signaling molecules anchored to or associated with lipid rafts. In resting B cells, BCRs are separated from lipid rafts and there is little affinity between them (72). The binding of antigens to BCRs which induces a transient ezrin dephosphorylation leads to a detachment of lipid rafts from the actin cytoskeleton, and promotes the interaction between BCRs and lipid rafts (18, 73) where downstream molecules such as Src family kinases are anchored (72). Soon after the transient dephosphorylation, there is a rephosphoryation of ezrin, leading to the reassembly of the actin cytoskeleton (53). Reassembly of the actin cytoskeleton stabilizes the interaction between BCR and other signaling molecules through trapping and stabilizing the raft-localized signaling complex (53, 72).

Besides, downstream signaling molecules can interact indirectly with the actin cytoskeleton and the recruitment of these molecules to BCR clusters can be promoted by actin associated proteins. For example, Grb2, the BCR signaling adapter, can be recruited through WASP, and promotes BCR signaling (88). The recruitment of molecules through actin cytoskeleton also down-regulates BCR signaling. As we have discussed earlier, BCR central-cluster formation is accompanied with the attenuation of BCR signaling. The molecular mechanism underlying this process has not been clearly understood, but a possible explanation is that actin regulators or adapters promote the inhibitory signaling molecules being collected to BCR clusters. From our unpublished data, it was found that during BCR signaling attenuation, neural WASP (N-WASP), an actin nucleation promoting factor, and the actin adapter protein Abp1 are recruited to BCR microclusters. Abp1 was found negatively regulate T-cell signaling through recruiting HPK1, a negative signaling molecule, to the immune synapse of T cell (89). Besides, N-WASP was found promoting the localization of SHIP to F-actin in poxviruses (90). To conclude these findings, it's supported that actin regulators involved in the signaling attenuation stage are likely to promote inhibitory signaling-molecule recruitment and down-regulate signaling.

## BCR Signaling Regulates Actin Remodeling

**Figure 1:**
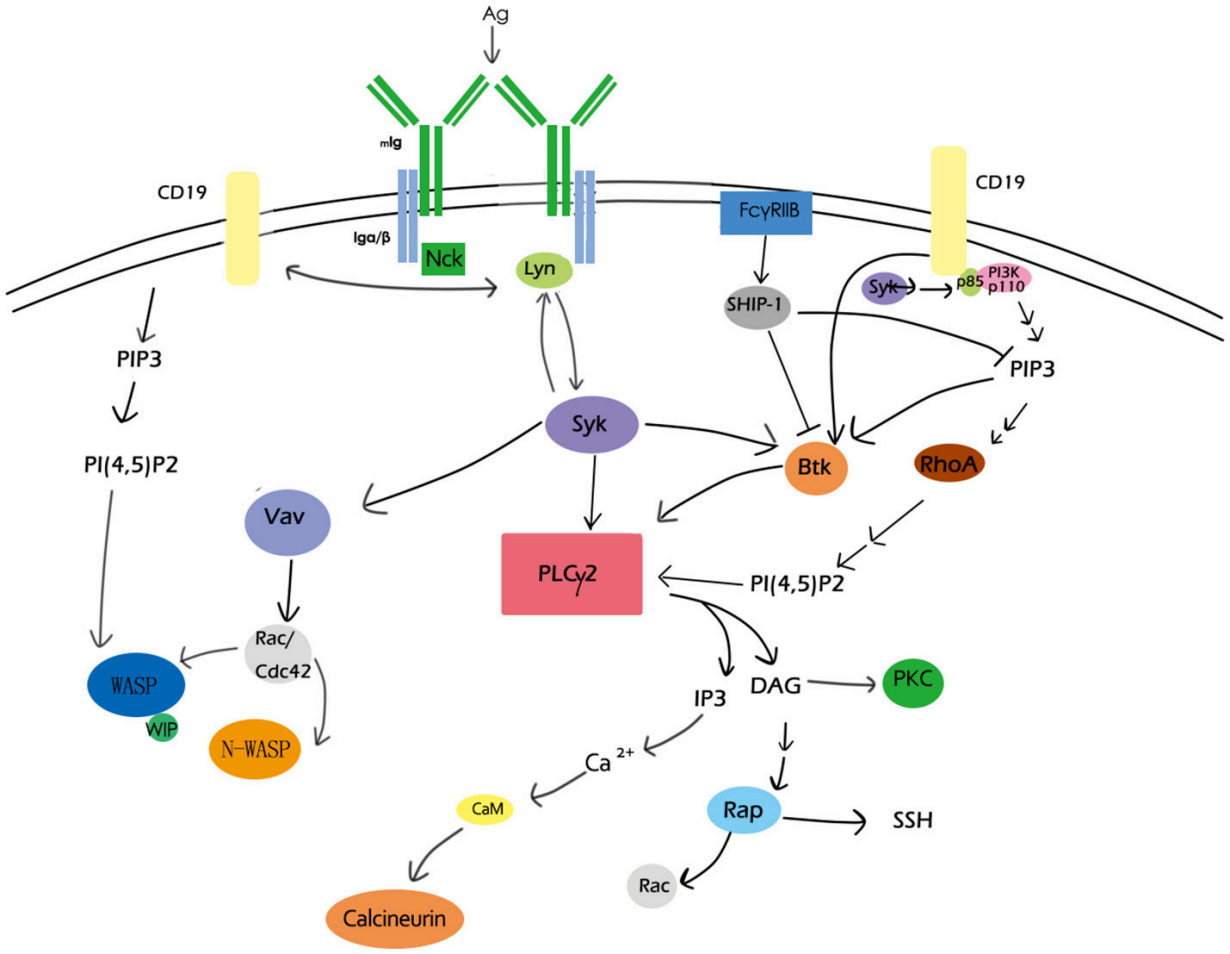
Overview of BCR signaling molecules involved in actin remodeling. CD19, PIP2, PLCγ2,PKC, the Rho family, and Rap GTPase, Btk, calcium, and WASP are major BCR signaling molecules involved in actin remodeling. These signaling molecules as well as their regulators form a network to participate in actin-cytoskeleton reorganization during B-cell activation.

During these processes of B-cell activation, the actin cytoskeleton undergoes dynamic, directional, and coordinated reorganization, which needs to be precisely regulated in response to extracellular clues. The polymerization of actin has been detected where the formation of BCR microclusters take place (41, 60, 74), and tyrosine kinase inhibitors could block actin remodeling induced by antigen stimulation (51), suggesting the regulation of the actin cytoskeleton actually depends on BCR signaling (Figure 1).

### BCR Signaling Regulates the Disassociation of Actin Cytoskeleton From the Plasma Membrane

**Figure 2:**
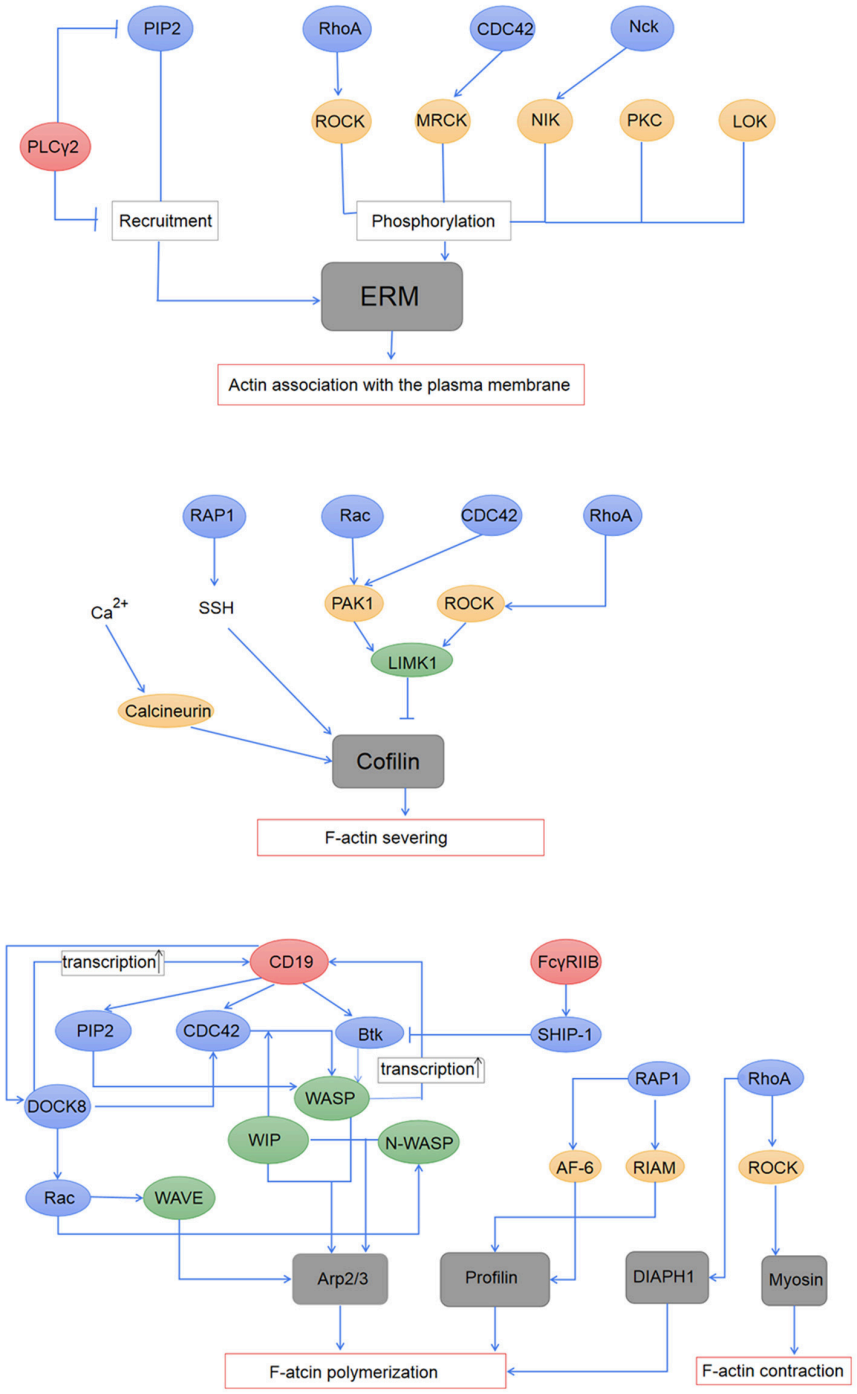
Regulation of BCR signaling on the actin cytoskeleton. The association of the actin cytoskeleton with the plasma membrane is mediated by activated ERM proteins. The ERM proteins are first recruited to the plasma membrane by PIP2, and then phosphorylated by PKC, LOK, and effector proteins of RhoA, CDC42, and Nck. PLCγ2 induced inactivation of the ERM proteins through its down-regulation on PIP2. Activation of cofilin induces F-actin severing, which is regulated by the Rho family and Rap1 GTPase, and also intracellular calcium. BCR signaling regulates actin polymerization mainly through the actin-nucleation promotion factor WASP and WAVE, both of which can promote the nucleation effect of Arp2/3. Profilin and DIAPH1, which are regulated by RAP1 and RhoA, respectively, are suggested to participate in actin polymerization during B-cell activation. BCR signaling also influences contraction of the actin cytoskeleton through the regulation of RhoA on myosin.

BCR signaling firstly induces the disassociation between the cortical actin and the plasma membrane through the regulation of the ERM proteins (53, 73, 91). ERM proteins interact with the plasma membrane through a common FERM region within the N -terminal domain and bind to F-actin through the actin-binding domain within the C terminus (42, 92). The phosphorylation of the critical threonine residue in the C terminal domain of ERM proteins induces the opening and exposure of the FERM structure and the actin-binding domain, and enables ERM proteins binding to both cortical actin cytoskeleton and plasma membrane. While the dephosphorylation in this domain leads to a closed conformation which both of the N- and C- terminal ends are engaged in an intramolecular association (93, 94), and results in ERM proteins uncoupling with F-actin and the plasma membrane. The conformational changes are controlled by phospholipids and kinases-mediated phosphorylation (29, 91). PIP2 promotes the recruitment of ERM to the membrane and the exposure of threonine residues within the C terminal domain which are phosphorylated by other signaling protein kinases including myotonic dystrophy kinase-related Cdc42-binding kinase, Rho-associated protein kinase (ROCK), protein kinase C, Nck interacting kinase, and lymphocyte-oriented kinase (LOK) (95–100). Activation of PLC γ which transforms PIP2 to IP3 induces the closed conformation of ERM proteins and disassociation of ERM proteins from the plasma membrane (101). Decreased level of PIP2 resulted from increased PLCγ activity was found enough to induce ERM dephosphorylation (102) (Figure 2). The following model of the regulation of ERM proteins during B-cell activation is suggested: during B cell activation by antigen stimulation, BCR signaling firstly induces a transient dephosphorylation of ERM, which increases the mobility of BCRs and lipid rafts by the disruption of spatial confinements (53). Followed BCR clustering and interaction with lipid rafts amplify tyrosine phosphorylation, and the continuous BCR signaling leads to rephosphorylation of ERM (53).

### BCR Signaling Regulates F-Actin Severing

The transient dephosphorylation of ezrin is accompanied with a decrease of F-actin induced by actin severing proteins (58, 72). The severing of pre-existing F-actin produces barbed ends and enough actin monomers for the formation of new branches of F-actin. Cofilin, one of the actin-severing proteins, takes a major part in actin severing during the activation of the B cell (58). In resting B cells, its actin-binding activity is inhibited due to phosphorylation of the serine 3 residue. The dephosphorylation at this region through Slingshot phosphatase (SSH) (103), which is inhibited by 14-3-3 protein-mediated sequestration, leads to the activation of its F-actin severing ability. BCR signaling molecules including GTPase Rap1 and the Rho family are suggested to participate in regulating cofilin activity. RhoA can inhibit the activity of cofilin through ROCK1 which activates LIM domain kinase 1 (LIMK1) which directly phosphorylates the ser 3 residue (104), while Rac and CDC42 activate the kinase PAK1 which also induces the phosphorylation of LIMK1 (105). In the contrast, GTPase Rap1 has been found to directly promote cofilin dephosphorylation (58). The mechanism underlying cofilin dephosphorylation induced by Rap has not been made clear. Since cofilin is phosphorylated mainly by LIMK1 which is not reduced by BCR signaling, the activation of cofilin may be a result of increased activity or release of SSH through various effector proteins of Rap1 GTP (58). Studies have proved that during B cell activation, the regulation of Rap on cofilin is crucial in B-cell spreading and BCR-microcluster formation, and also in the regulation of both actin and microtubule network at the immune synapse (58, 85). Besides, it is showed that dephosphorylation of cofilin relies on cytoplasmic Ca^2+^ in different types of cell (105, 106). Increased level of intracellular Ca^2+^ promotes cofilin activation through direct or indirect interactions with the calcium-dependent phosphatase calcineurin. In B cells, the level of cytoplasmic Ca^2+^ has been found to directly correlate with the generation and disruption of the protrusive actin structures, and was implicated indispensable in both B cell adhesion and spreading to antigen-presented surface (106). The increase of Ca^2+^ induces the depolymerization of F-actin in the membrane protrusions, while the sequestering of Ca^2+^ leads to the growth of F-actin. The regulation of Ca^2+^ on cofilin may be one of the molecular mechanisms underlying the link between cytoplasmic Ca^2+^ and actin dynamics (106, 107) (Figure 2).

### BCR Signaling Regulates Actin Polymerization

Regulation of BCR signaling on actin polymerization is mainly mediated by the actin-nucleation promotion factor WASP and WASP-family verprolin homologous protein WAVE (43, 78, 108) which are directly regulated by the Rho family GTPase (109). WASP binds to and activates Arp2/3, and thus induces actin polymerization (110). WASP contains the CDC42/Rac-interactive (CRIB) and the C-terminal verprolin homology/cofilin-homology/acidic (VCA) region. The two regions interact with each other and lead to a basely inactive auto-inhibitory conformation of WASP (110), and the binding of WIP to the WASP homology 1 region (WH1) stabilizes this inactive conformation (23, 24). When BCR is stimulated, activated CDC42 binds to the CRIB region and PIP2 combined with the basic region of WASP, inducing conformational changes. The changes allow the conserved tyrosine and serine of WASP be phosphorylated by the Src family kinases, which can further stabilize its open conformation. Opening and activated WASP binds to G-actin and Arp2/3 via the VCA region, and leads to the branching of F-actin (110). WASP deficient B cells showed impaired formation of central clusters, internalization of antigen and increasing BCR signaling (60). Additionally, N-WASP which is 50% homologous with WASP also functions in B cells (111, 112). Studies showed that deficiency of both WASP and N-WASP resulted in more severely disrupted B cell spreading and BCR microcluster formation compared with WASP-deficient only B cells (113, 114). However, the influence on the amount of F-actin was opposite in WASP and N-WASP-deficient B cells, suggesting redundancy but also distinct functions of WASP, and N-WASP (113). The binding of WIP stabilizes WASP and protects it from degradation, and thus participates in actin reorganization. Besides, WIP directly binds to and promotes polymerization of F-actin in a WASP-independent way (81, 115). It was found that binding of WIP to actin influences CD19 diffusion through the regulation of CD81 expression (116, 117). In T cells, WIP acts as a bridge to bring dedicator of cytokinesis protein 8 (Dock8), a GEF for CDC42 to WASP and actin, and may be another mechanism for the regulation of WIP on the actin cytoskeleton in B cells (118).

BCR signaling molecules Btk and SHIP-1 play an important role in WASP regulation. During B cell activation, Btk is recruited to the plasma membrane and is phosphorylated, which requires PI3K activation (CD19 signaling pathway), and kinase Lyn or Syk (119, 120). Phosphorylated Btk later activated PLCγ2 and triggers Ca^2+^ signaling (20, 61). Besides, Btk acts as the scaffold which brings PIP5KI to the plasma membrane and thus leads to the production of PIP2 (121, 122). Btk has been confirmed as an important signaling molecule in promoting WASP activation via Vav and PIP2 (123) or through direct interaction with WASP (124). It was found that Btk is indispensible in BCR cluster formation and B cell spreading. The activation of Btk is inhibited by SHIP-1 (120, 125), and participates in BCR central cluster formation and BCR signaling attenuation (60). There exists a balance between CD19-Btk and FcγRIIB-SHIP mediated signaling (60). Abnormal changes of the signal strength is concerned with immunological diseases. It has been found that BCR signaling molecules including Dock8, Mst1, and WASP positively regulate *cd19* transcription (116, 126, 127). Deficiency of these proteins all leads to decreased CD19-Btk signaling, which results in reduced BCR clustering and B cell spreading on antigen-associated membrane. These findings provide with new mechanisms for the symptoms of immunodeficiency diseases (116, 117, 126–128).

The WAVE complex is combined of five subunits in B cell, including specifically Rac-associated protein 1 (Sra1), Nck-associated protein 1-like (NCKAP1L), ABL interactor 1 (ABI1), WAVE2, and hematopoietic stem/progenitor cell protein 300 (HSPC300) (129). This complex undergoes changes from inactive to active state during the stimulation of BCR. When stimulated, the binding of Rac-GTP to Sra1 of the WAVE complex potentially induces the conformational changes and permits access of WAVE to the Arp2/3 complex and G-actin, which leads to actin polymerization, and thus the WAVE complex participates in the formation of membrane protrusion during B cell mobility (130, 131). The Rac-GTP contains Rac1 which is universally expressed and Rac2 which is expressed only in the hematopoietic system. Both of them are activated during BCR signaling. It was found that Rac2 rather than Rac1 plays an important role in both B-cell adhesion to ICAM-1 and immune-synapse formation during B-cell activation, and Rac2-deficient B cells exhibit impaired actin polymerization (43). Rac1 and Rac2 can compensate each other to some extent, but either of their deficiency can lead to failure in B-cell maturation (132). The different functions as well as the redundancy between these two Rac proteins need further studies.

Other proteins involved in BCR signaling mediated actin polymerization include profilin which can be recruited through GTP Rap1 effector proteins RIAM and AF-6 (22). The Rho family member RhoA is suggested to promote actin polymerization through its effector diaphanous homolog 1 (DIAPH1) which takes a part in regulating dynamics of F-actin (133). Besides, RhoA regulates the flow of F-actin through ROCK which increases levels of phosphorylated myosin light chains (MLCs) (134) which binds to and stimulates contraction of the ends of F-actin (Figure 2).

## Concluding Remark

*In vivo*, B cells are activated mainly by membrane-associated antigens which differ in various properties including density, distribution, mobility, valence as well as the topography and stiffness of the presenting membrane, and thus require exquisite regulation to adjust to different environment. The cooperation between BCR signaling and the actin cytoskeleton is the mechanism underlying this innate regulation of B cells (71). During B cell activation, the actin cytoskeleton undergoes reorganization which is essential for changes in BCR mobility, B cell morphology and molecular interaction, and thus influences the formation of BCR microclusters and the immune synapse, which are important for BCR signaling and antigen accumulation. The dynamic of the actin cytoskeleton is in turn modulated by BCR signaling, and thus forms a feedback loop. In the network composed of BCR signaling molecules, different molecules may have similar effects on the cytoskeleton, while one molecule may have opposite functions through regulating different actin-binding proteins, and there exists regulatory relationship between these molecules, which makes it difficult to study the specific regulating mechanism underlying the entire process of B cell activation. Besides, it needs to be explored in the future whether or how the actin cytoskeleton participates in triggering BCR signaling initiation and in the distinct response to antigens of different B cell subsets, and how the actin cytoskeleton is influenced by the milieu of stimuli. Understanding of the cooperation between the actin cytoskeleton and BCR signaling will help us to find new mechanisms and targets in B-cell related immunological diseases.

## Author Contributions

CL and BR organized the article. JL and WY wrote the draft. YJ, DK, LY, JC, ZY, ZP, XL, YW, and XS revised the draft.

### Conflict of Interest Statement

The authors declare that the research was conducted in the absence of any commercial or financial relationships that could be construed as a potential conflict of interest.

## Nomenclature

Ag, antigen; BCR, B cell receptor; Btk, Brutons tyrosine kinase CD19, cluster of differentiation 19; Cdc42, cell division control protein 42; ERM, ezrin-radixin-moesin; F-actin, filamentous actin; FcγR, Fc gamma receptor; Igα, immunoglobulin α chain; Igβ, immunoglobulin β chain; IgM, immunoglobulin M; IgG, immunoglobulin G; ITAM, immunoreceptor tyrosine-based activation motif; Lyn, LYN proto-oncogene; Src family tyrosine kinase; mAg, membrane-associated antigen; MHC, major histocompatibility complex; Erk, phosphorylated extracellular regulated protein kinases; PKC, protein kinase C; PI3K, phosphatidylinositol 3-kinase; PLCγ, phospholipase C gamma 2; pSHIP, phosphorylated SH2-containing inositol phosphatase; Syk, spleen tyrosine kinase; TIRFm, total internal reflection fluorescent microscopy; TCR, T cell receptor; Vav, vav guanine nucleotide exchange factor; WASP, Wiskott-Aldrich syndrome protein.
